# One Health and Antibiotic Resistance in Agroecosystems

**DOI:** 10.1007/s10393-018-1324-7

**Published:** 2018-03-14

**Authors:** Lisa M. Durso, Kimberly L. Cook

**Affiliations:** 1 USDA, ARS, Agroecosystem Management Research Unit, 251 Filley Hall, UNL East Campus, Lincoln, NE 68583 USA; 2 USDA, ARS, Bacterial Epidemiology and Antimicrobial Resistance Research Unit, 950 College Station Rd., Athens, GA 30605 USA

**Keywords:** Antibiotic Resistance, Agriculture, One Health, Agroecosystems, Food, Farms, Environment

## Abstract

Agriculture reflects One Health principals, with the job of the farmer being to sustainably balance human, animal, and soil health. It is imperative to include an agricultural perspective when addressing antibiotic resistance (AR) from a One Health perspective, as the farmers, ranchers, and agricultural professionals have an intimate working knowledge of these complex systems, and they will be on the front lines of implementing on-farm control measures. Currently, communication across the One Health triad (humans, animals, environment) regarding agricultural AR is hindered by ambiguous language, complicated by cultural and linguistic differences that can lead to the conclusion that the other participant is not aware of the facts, or has ulterior motives. This work explores and identifies the language and vocabulary of AR in the context of supporting strategic short- and long-term problem solving in a One Health context.

*“If you eat, you are involved in agriculture.”* Wendell Berry (1990)

The complexity of the antibiotic resistance (AR) problem and its widespread impacts for human, animal, and environmental health make it “the quintessential One Health issue” (Robinson et al. 2016; Lammie and Hughes 2016); however, the environmental dimensions of One Health have historically been underrepresented (Barrett and Bouley 2015). Agriculture is, at its core, an embodiment of One Health principals, with the job of the farmer being to sustainably balance human, animal, and soil health. While these principles are a well-known part of the narrative for North American organic livestock and poultry farms, they are also an ingrained part of the conventional farmers’ principles of farm–animal–family stewardship (Sullivan et al. 1996). In the context of AR, there is broad consensus that the One Health community needs to reduce transfer of antibiotic drugs, AR bacteria, and antibiotic resistance genes (AR genes) to, and through, the environment (Berendonk et al. 2015; Williams-Nguyen et al. 2016; Allen et al. 2010; Pruden et al. 2013). This consensus arises from and is coupled to the growing body of knowledge on environmental inputs of drugs, bacteria, and genes from organic (Sutherland et al. 2013; USDA 2006), natural (Syrengelas et al. 2017), and conventional food animal production (Marti et al. 2014; Cook et al. 2014; Brooks et al. 2014; McKinney et al. 2010; Sandberg and LaPara 2016; Chambers et al. 2015; Qiao et al. 2018).

From the human health perspective, it is the acquisition of multiple resistances by pathogens that is the primary concern. From an environmental perspective, the soil is a reservoir of AR and the original source of AR in the clinic (Fosberg et al. 2015). The evidence needed for causal links required to assess current and emergent AR health risks from agroecosystems remains a knowledge gap (Durso and Cook 2014; Williams-Nguyen et al. 2016; Topp et al. 2018; Franklin et al. 2016).

## One Health Terminology

*“If I had only one hour to solve a problem, I would spend up to two-thirds of that hour in attempting to define what the problem is.”* Finley and Ziobro (1966)

The One Health community is composed of many disciplines, each with their own culture, language, and conceptual framework. One challenge in addressing AR is acknowledging that public health, animal health, and agroecosystem professionals use similar terms, but define them differently. Language shapes our thoughts, influences how we understand cause and effect, and impacts how groups assign blame. (Agar 2006; Fausey and Boroditsky 2010; Fausey and Matlock 2011; Thomas and McDonagh 2013). Research shows effective problem solving requires identifying terms where the participating groups have differences of interpretation, sometimes termed “rich points” (Lederach 1991; Wilkinson and Eidinow 2008; Agar 2006). These differences are often attributable to specific associations and connotations that are characteristic of individual disciplines. Ambiguity of terms across disciplinary cultures hinders strategic communications (Ramirez et al. 2008; Wilkinson and Eidinow 2008) and, at the extreme, may lead to conclusions that the other participant has made an error, is not aware of the facts, or has ulterior motives (Agar 2006). An unambiguous shared vocabulary supports rigorous reasoning and is the foundation for strategic transdisciplinary problem solving (Thompson 2009; Groysberg and Slind 2012; Knapp et al. 2015).

One example of a “rich point” in the One Health AR sphere is the term “resistance.” In clinical settings, resistance is always linked to a pathogenic bacterial isolate and the threat to human or animal health is direct (Berendonk et al. 2015). Clinical resistance is measured by standardized assays to determine minimum inhibitory concentrations of an antibiotic necessary to achieve positive treatment outcomes for specific disease-causing bacteria (CLSI 2017). In contrast, most agricultural and environmental bacteria are not pathogens, the term “resistant” can be applied either to an isolate, or to a whole community of bacteria, and the threat to health is indirect (Williams-Nguyen et al. 2016). There is no consensus or standardized methods for defining or determining AR in non-clinical settings (Berendonk et al. 2015; McLain et al. 2016; Durso and Schmidt 2018), and the detection of any part of any target gene or determinant DNA sequence results in the sample being described as “resistant.” Where possible, researchers use clinical standards, but the relevance of those methods and links to risk (i.e., disease outcomes) are not known for most environmental bacteria (Williams-Nguyen et al. 2016; McLain et al. 2016). Though cumbersome, the use of qualifiers such as “clinical resistance” or “environmental resistance” would provide vocabulary to distinguish the two concepts when working in a One Health setting.

As a second example, the term “environment” itself has different common uses in the human health community compared to the environmental health community, particularly in the context of AR. In human health settings, “the environment” is generally conceptualized as anything outside of the patient and is usually used in the context of the patient room, or other hospital settings (Cheng et al. 2018). This contrasts with the broader, agriculturally relevant definition of “the environment”—defined as soil, water, and air (Durso and Cook 2014; Berendonk et al. 2015). Here, qualifiers such as “built environment” and “natural environment,” while not completely accurate in all instances, may provide a useful means of clearly communicating across the One Health triad.

In a final example of how the adoption of more precise language is essential to the effective communication needed for a One Health approach, it is common, particularly when discussing antibiotic use in food production, to attribute agricultural AR to the “overuse,” “misuse,” “unnecessary use,” and “abuse” of antibiotics (Jørgensen et al. 2017; Jia et al. 2015). However, *any* use of antibiotics can select for resistance. According to the CDC (2013), even prudent use of antibiotics still provides selective pressure for development of resistance. The difference between “overuse” and “use” is an implied message of blame, and of a known solution—that if only the drugs were used properly, resistance would not be a problem. This imprecision in language results in a subtle but real barrier hindering strategic communication across disciplines and masks a growing body of evidence suggesting prudent use is necessary, but not sufficient to address AR in agroecosystems (Seiler and Berendonk 2012).

## Drugs, Bacteria, and Genes in the Environment

*“When you can measure what you are speaking about … you know something about it.*” William Thomson aka Lord Kelvin (1889)

When evaluating AR in the environment, it is essential to clearly convey which AR component is being measured and discussed; drugs, bacteria, or genes (Durso and Cook 2014). It is common to measure a small set of targets, and then talk about the results using the general term “antibiotic resistance.” However, it is clear that different drug classes, and their associated AR bacteria and genes, each have their own ecologies (Durso et al. 2016). The lack of resolution in terminology can cause confusion and hinder strategic communication. As an example, imagine discussing the impacts of food on human health, when, regardless of what category of food was evaluated, the strategic and policy discussions evaluated the outcomes only at the level of “food.”

Of note, antibiotic drugs and their bioactive breakdown products, though not technically considered “antibiotic resistance” themselves, are widely considered the primary driver of AR (Subbiah et al. 2016), and discussions seeking to address agricultural and environmental resistance are frequently focused on the presence of anthropogenically controlled drugs in environmental matrices (Ashbolt et al. 2013; Subbiah et al. 2016; Aga et al. 2016; Amarakoon et al. 2016). There are 183 drugs listed as critically important for human medicine by the World Health Organization (WHO 2016), each carried by a multitude of bacteria, and coded for by hundreds of genes. Precision when discussing these targets is essential, because the choice of target can influence the conclusions that are being drawn about resistance in a study. For example, in a survey of tetracycline genes in native prairie soils 50% of the prairies were positive for *tet*(B), compared to 95% positive for *tet*(D). Different conclusions would likely be drawn if *tet*(B) were used to assess resistance compared to *tet*(D) (Durso et al. 2016).

Another “rich point” in One Health AR discussions is the role of antibiotic drugs as a driver of AR. The relationships between antibiotic drugs, AR bacteria, and AR genes in agroecosystems are complex. Sometimes, clear correlations are observed between drugs and resistance; this is the outcome that is generally expected. In the presence of more drugs, more resistance is observed (as measured by an increase in the number or types of AR bacteria and/or AR genes) (Knapp et al. 2010; Sancheza et al. 2016; Österberg et al. 2016). However, the presence of more drugs does not always correlate with more resistance in environmental settings (Ghosh and LaPara 2007; Dalkmann et al. 2012; Mantz et al. 2013; Jechalke et al. 2015).

Unlike assessments done in animals (Dorado-García et al. 2016), the complexities of natural systems confound efforts to identify direct causal relationships in manure, soil, water, and air (Franklin et al. 2016; Williams-Nguyen et al. 2016). Heavy metals, biocides, detergents, and iron deprivation also co-select for resistance genes (Seiler and Berendonk 2012). AR genes located on mobile elements are enriched indirectly via selection for other genes located on the same replicon (Alonso et al. 2001; Gillings et al. 2008). In addition, evidence suggests a role for antibiotics beyond simply inhibiting bacterial growth. Some bacteria use antibiotics as a carbon source (Dantas et al. 2008), and transcriptomics reveals the potential for antibiotics to serve as a signaling molecules, influencing stress response, motility, and biofilm formation (Romero et al. 2011). Thus, there are many possible functions of environmental bacteria that can potentially impact selection of resistance.

In addition to identifying terminology that has multiple definitions across the One Health partners, it is also useful to acknowledge and articulate the outcome of interest in environmental AR discussions. For example, given that AR occurs naturally in soil, and as a result of historical and current anthropogenic activities, does source of the target matter? The answer to this question depends on the outcome of interest. From the perspective of reducing risk, and reducing the occurrence of untreatable infections in humans and animals, the answer is no. Regardless of how the AR bacteria or AR genes got into the agroecosystem—whether naturally occurring or the result of anthropogenic activities—the goal is to limit their transfer out of the system. Knowing the background and baseline levels of resistance is important for measuring the impact of management and mitigation practices, but it is not mandatory to source-track individual bacteria or genes. In contrast, when assessing agricultural AR risk from a regulatory perspective, it is critical to distinguish between AR bacteria and AR genes that are the result of current anthropogenic activities, from AR bacteria and AR genes that occur naturally, or that were part of the baseline resistance on a farm. Beginning discussions by clarifying goals is another technique that can improve One Health teams.

Complexities of the agroecosystem environment continue to confound efforts to define risk associated with AR across the farm to table continuum. Continuing efforts to harmonize the lexicon and to frame risk and define relevant outcomes across the branches of One Health professionals will inform and strengthen One Health efforts.
